# Scottish Vision Group Meeting, Isle of Arran, Scotland, 10–12 March 2017

**DOI:** 10.1177/2041669517709809

**Published:** 2017-05-24

## Keynote 1: fMRI Recording From the Human LGN – Four Unexpected Findings

**Kathy T. Mullen, Dorita F. Chang and Robert F. Hess**

McGill University, Montreal, Canada

### 

 Functional magnetic resonance imaging allows direct recording from the functioning human LGN, whereas previously our understanding of this primary thalamic nucleus was based on single cell recordings or lesion experiments in primates. FMRI studies, however, have thrown up some surprising results that initially appear to be at odds with data from the previous approaches. Here, I discuss some of these results from my lab, focusing on four different issues: (a) the responses to colour in the LGN as compared with these responses to the same stimuli in V1; (b) the effect of Amblyopia on human LGN and cortical responses and V1; (c) the responses to temporal frequency in the LGN and V1; and (d) the differential adaptation in the LGN for coloured and achromatic stimuli. Overall, I will argue that these results suggest that fMRI recordings are as much (or more) influenced by the descending cortico-thalamic feedback as by the ascending driver neuron responses. If this is correct, fMRI recording of the LGN can provide a very different perspective on its role in human visual processing.

## Keynote 2: Ocular Dominance Plasticity in Humans

**Robert F. Hess**

McGill University, Montreal, Canada

### 

Hubel and Wiesel, Nobel Laureates in 1981, were the first to discover that columns exist in the visual cortex representing left and right eye inputs (*ocular dominance columns*), and they also found that there is a *critical period for visual development* that occurs within the first year of life. More recently, however, it has become clear that some plasticity remains into adulthood. My recent work shows that this plasticity extends to ocular dominance in adults, which could potentially lead to direct therapeutic benefit. We have shown that short-term visual deprivation in adults improves sensitivity of the deprived eye and reduces sensitivity of the nondeprived eye, allowing the two eyes' inputs to be rebalanced at the level of binocular integration. In this talk, I will review the evidence using a variety of techniques.

## The Output of VIP-Interneurons in the Primary Visual Cortex Is Best Reflected by Presynaptic Activity, Not Somatic Activity

**Rozan Vroman and Leon Lagnado**

School of Life Sciences, University of Sussex, Brighton, UK

### 

Recent advances in two-photon calcium imaging in awake mice have made it possible to study the effect of different behavioural brain states on V1 circuitry. Many studies assume that somatic activity can be used as a measure for neuronal output. We set out to test the validity of this assumption by comparing somatic activity of VIP-interneurons in Layer 2/3 of V1 with their presynaptic activity. In our approach, we used mice expressing genetically encoded calcium indicators in VIP-interneurons across the whole cell (VIP:GCaMP6f) or confined to presynapses (VIP:SyGCaMP5; Dreosti et al., 2009, *Nature Methods*, *6*, 883–889). Mice were exposed to a visual stimulation protocol consisting of minute-long presentations of moving Gabor gratings (0.04 cycles per degree, 2 Hz) alternated by half a minute of grey screen. During imaging, mice were placed on an air-suspended Styrofoam ball, allowing them to run voluntarily. We compared the activity both outwith and during visual stimulation, and with and without locomotion. VIP-synapses showed stronger suppressive responses to the visual stimulus than VIP-somas. Furthermore, VIP-somas were mostly positively correlated with locomotion, whereas in VIP-synapses, we observed a roughly equal split between positive and negative correlations. Focussing on the interaction between locomotion and visual stimulation, the excitatory effect of locomotion in VIP-somas increased over the course of the visual stimulus. This property was shared only with the positively correlated VIP-synapses. Our results suggest that when aiming to make statements about the involvement of VIP-interneurons in V1 Layer 2/3 circuitry, it is crucial to measure from synaptic terminals instead of somas.

## Modelling Response Variability in Disparity-Selective Cells

**Sid Henriksen and Jenny Read**

Institute of Neuroscience, Newcastle University, Newcastle upon Tyne, UK

**Bruce Cumming**

Laboratory of Sensorimotor Research, National Institutes of Health, Bethesda, MD, USA

### 

Stereo vision relies on disparity-selective cells in primary visual cortex. The binocular energy model (BEM) has been successful in capturing a range of properties of these neurons. However, the response variance of these model units increases with the square of the mean firing rate. Importantly, this variance is stimulus driven, reflecting the effect of different images while disparity is fixed. Recording from V1 neurons in the macaque, we used a two-pass method to separate stochastic variability (‘internal variance’) from stimulus-induced variability (‘external variance’). We found that V1 neurons’ variance generally only change with the mean, and not its square. This failure is partly due to the BEM’s highly constrained structure. We fit more general linear–nonlinear models, with the number of subunits as a free parameter, to neuronal data. This general architecture is able to capture both the mean and variability of real cells much better. However, problems remain. We show that while the new model captures the ‘internal’ variability due to the spike generation process, it underestimates the ‘external’ variability produced by different random noise patterns with a given correlation and disparity. The new model also shows a relationship between the Fano Factor (Variance/Mean) and the mean which we have reported previously in the BEM, and which is absent in real cells. Thus, this substantial generalization of the BEM is still not an accurate model of real V1 neurons.

## Stimulus Precision Matters: High Precision Point-Light Stimuli Reveal Novel Processing of Biological Motion

**Lawrie S. McKay**

School of Psychology, University of Glasgow, Glasgow, UK; Netherlands Institute for Neuroscience, Amsterdam, Netherlands; Research Imaging Centre, Ninewells Hospital & Medical School, Dundee, Scotland; School of Media, Culture and Language, University of the West of Scotland, Paisley, Scotland

**Lee Seul Shim, Salim Al-wasity**

School of Psychology, University of Glasgow, Glasgow, UK

**Ulf Ahlstrom**

FAA Technical Centre, William J. Hughes Technical Center, Atlantic City, USA

**Frances Crabbe**

Institute of Neuroscience & Psychology, University of Glasgow, Glasgow, UK

**Frank E. Pollick**

School of Psychology, University of Glasgow, Glasgow, UK

### 

Biological motion perception is often studied using point-light displays (PLDs). Two methods exist for generating these displays: a digitised method where actors are videotaped with markers placed on the joints and a motion capture (MOCAP) based system. These create stimuli of low and high precision, respectively (LP and HP). To date, nobody has attempted to determine whether these different levels of stimulus precision differentially activate biological motion processing regions. We tested this by recreating a widely used set of actions using the high-precision MOCAP method and then degraded them to match the lower precision of a digitised dataset. We then ran identical experiments on the same subjects contrasting intact and scrambled versions of the PLDs. We found that at the group level, left pSTS was found only using the LP stimuli. Furthermore, using the HP stimuli, we found that regions typically associated with action understanding responded much more strongly to scrambled than intact displays. Closer inspection revealed that the differences were present in the LP experiment, but were smaller than with the HP stimuli, suggesting that this difference may have been missed in previous research as a result of low stimulus precision. Furthermore, those region of the action understanding circuit more activated by scrambled displays suggest that our understanding of its functions may need to be reconsidered. We speculate that this circuit is engaging in automatic internal simulation of discrete body parts more than coherent form, and that predictive coding errors may provide a plausible mechanism to explain this difference.

## How Does Zebra Colouration and Herd-Size Influence Performance in a Video-Game Tracking Task?

**P. G. Lovell**

University of Abertay, Dundee, Scotland

**N. E. Scott-Samuel**

University of Bristol, Bristol, UK

### 

The adaptive advantage of zebra colouration is a matter of considerable debate. One plausible hypothesis is that zebra stripes amplify the ‘confusion effect’ (reduced predation risk attributable to increased difficulty in targeting and capturing an individual target in a large aggregation of prey). We implemented a video game task where participants (*n*=20) attempted to intercept a particular zebra – by colliding with it – while we manipulated the colouration of the herd. The speed and scale of the virtual zebras approximately matched those of real zebras and their motion trajectories were controlled by a 2D version of the boids algorithm, which simulates flocking in birds. We manipulated herd size (1, 16, 32 items) and colouration (zebra vs. horse). We replicated the confusion effect: Participants collide more often with the incorrect target as herd size was increased. We also measured the extent to which participants successfully point themselves at the target animal throughout their motion paths. Target animals are less likely to be centrally located in the screen when the set-size was larger: the absolute mean visual eccentricity (lion bearing relative to zebra location) was around 40° for a single target but this rose to around 55° when there were 32 targets. We found no additional benefit for zebra colouration.

## Visual Awareness of Familiar Faces

**Kay Ritchie**

University of Lincoln, UK

### 

We know from a large body of research that there are differences between the ways familiar and unfamiliar faces are processed in the brain. To date, however, there has been only a limited exploration of how the high-level concept of our familiarity with a person may influence low-level processes such as visual awareness. Faces have been used previously in binocular rivalry paradigms to establish the effect of facial expression on the relative dominance of percepts. This study investigates the effect of familiarity on the perceived dominance of percepts by presenting familiar and unfamiliar faces (holding expression constant) in a binocular rivalry paradigm. Face/house rival pairs comprising a house and either a familiar or unfamiliar face are presented via a mirror stereoscope. Three experiments will be discussed: within-subjects design (½ faces familiar), between-subjects design (faces familiar to ½ participants) and mixed design (unfamiliar, newly learned, famous and personally familiar faces). This research has important implications for understanding how the visual brain processes familiar people and how we learn new identities.

This research is funded by a University of Lincoln College of Social Science research grant.

## Visuospatial Working Memory for Competing Emotional Faces

**Marlene Poncet**

School of Psychology, University of Aberdeen, UK

**Sara Spotorno**

School of Psychology, University of Glasgow, UK

**Margaret C. Jackson**

School of Psychology, University of Aberdeen, UK

### 

During social interaction, we use working memory to continuously keep track of the identity and the position of the people around us. Previous work in our lab has shown that using homogeneous displays of happy or angry faces, working memory for identity-location binding was better for happy than angry faces. In this study, we investigated how differing emotional faces compete at encoding to influence identity-location binding. To do this, participants were shown four faces of different identities at random screen locations for 6 s. Two faces carried one emotion while the two other faces carried a different emotion (selected from happy, angry, sad and fearful – all possible pair combinations were used). After a blank maintenance interval of 1 s, one of the four faces was presented at the centre of the screen with a neutral expression. Participants had to relocate this face to where it was first presented, matching the correct identity with the correct location. Although emotion was not relevant for the task, we found higher correct relocations for happy faces than for other emotional faces, replicating our previous work using homogenous emotion displays. Further, sad and angry faces were generally better remembered when they were paired with happy faces than when they are paired with fearful faces. Thus, identity-location binding for happy faces seems to be more efficient and less resource intensive than for the other emotional faces.

## Initiating Both Joint and Nonjoint Attention Improves Memory for Other Race Faces

**Bert Timmermans, Dania El Amin and Chris Luke**

University of Aberdeen, Aberdeen, UK

### 

The own race bias (ORB) is an extremely well-documented phenomenon in face perception, showing that under a variety of circumstances, people have better post-learning recognition of their racial ingroup than racial outgroup faces (Hugenberg et al., 2013, *Visual Cognition*, *21*, 1392–1417). Recently, Adams et al. (2010, *Journal of Experimental Social Psychology*, *46*, 478–481) showed that the ORB only exists for faces showing direct gaze, but not for averted gaze, where better memory for own-race faces disappears. However, it was recently shown (Pfeiffer et al., 2014, *NeuroImage*, *101*, 124–137) that Initiating Joint Attention (Mundy & Newell, 2007, *Current Directions in Psychological Science*, *16*, 269–274) activates the brain’s reward system, associated with increased liking. In two studies, we investigate whether anthropomorphic own-race and other-race avatars that either respond to participants’ gaze shift with joint attention or look the other way produce the ORB. Studies had participants interact with and memorise 20 own-race and 20 other-race faces. The interaction was such that participants had to choose to look at a target left or right of the face, and the face would either look directly at them (direct gaze condition), follow their gaze (joint attention condition) or look at the other target (nonjoint attention condition). In the first experiment, gaze contingency was manipulated between-participants, in the second experiment, only the JA and NJA conditions were tested within-participants. Results show that not just joint attention, but to a degree also nonjoint attention reduced the ORB, by improving the recognition of other-race faces.

## Is a Reduced Sensitivity to Facial Emotional Expressions Linked to the Severity of Parkinson’s Disease?

**Joanna Wincenciak and David J Burn**

Institute of Neuroscience, Newcastle University, Newcastle upon Tyne, UK

**Louise S. Delicato**

School of Psychology, University of Sunderland, Sunderland, UK

### 

People with Parkinson's (PwP) tend to have difficulty expressing and recognizing facial expressions. However, it is unclear precisely which expressions PwP find most difficult and what is the effect of disease severity on emotion recognition (e.g. Gray & Tickle-Degnen, 2010, *Neuropsychology*, *24*(2), 176–191). We characterized the ability of PwP to recognize emotions from facial expressions at the level of the individual using the psychophysical methodology. PwP performed the emotion discrimination task for upright and inverted facial expressions of happiness, surprise, disgust, anger, fear and sadness while they were on medication. For all participants and all expressions, performance increased from chance (50%) to 100% correct as intensity of expression increased. Fitted functions describing performance for happy and disgusted expressions were shifted to the left of other expressions for PwP, suggesting an increased sensitivity to happiness and disgust compared with other expressions. There appeared to be a difference in the spread of fitted functions linked to the disease progression: PwP showed a single cluster describing performance for all expressions; while healthy controls show two clusters. There was also a greater variability in the sensitivity to emotions in PwP, which might be linked to the severity of the disease. In addition, PwP and healthy controls showed a small reduction in sensitivity for the expression they were most sensitive to when it was inverted (face inversion effect). For PwP, there was a considerable face inversion effect for the expression they were least sensitive to, suggesting a disruption of configural face processing.

## The Effect of Autistic Traits on Bandwidth of Spatial Frequency Mechanisms Using Visual Masking

**Margaret H. Laurie**

University of Glasgow, Glasgow, Scotland; University of Edinburgh, Edinburgh, UK

**David R. Simmons**

University of Glasgow, Glasgow, Scotland

### 

The neural noise theory of Autism Spectrum Disorder (ASD) hypothesises increased internal noise in sensory systems of individuals with ASD, which could explain visual processing attributes of ASD (Simmons et al., 2009, *Vision Research*, *49*, 2705–2739). The bandwidth of sensory processing channels in ASD could be altered by neural noise (DeAngelis et al., 1995, *Proceedings of the National Academy of Science of the United States of America*, *92*, 9682–9686)*.* Bandwidths of spatial frequency channels were estimated using the following method; First, contrast thresholds were obtained at 0.5, 1 and 2 cycles per degree (cpd) using a standard Gabor stimulus. Then, thresholds for the 1 cpd stimulus were measured again, with the stimulus masked by another Gabor (either 0.5, 1 or 2 cpd) at a fixed contrast (10× dectection threshold). A threshold ratio, calculated by dividing masked by unmasked threshold at 1 cpd, was then used to estimate channel spatial bandwidth. Gabor envelope size was adjusted in each case to equalize stimulus spatial bandwidths, and carrier phase, for both mask and test stimulus, was either positive or negative sine relative to the envelope. A median split established groups of higher (*n* = 18) and lower (*n* = 20) scorers on the Autism Spectrum Quotient (AQ; Baron-Cohen et al., 2001, *JADD*, *31*(1), 5–17). Bootstrapped *t-*tests showed that higher AQ scorers had higher threshold ratios. This suggests that individuals with higher AQ scores have broader bandwidth in spatial frequency channels, and thus, potentially increased levels of neural noise or, alternatively, GABAergic imbalance (Pizzarelli & Cherubini, 2011, *Neural Plasticity, ID:* 297153).

## Migraine and the Motion Streak

**Louise O’Hare**

University of Lincoln, Lincoln, UK

### 

Migraine is a neurological disorder with strong links to vision, and those with migraine tend to show differences compared with controls on visual processing tasks. One of the most robust differences is poorer performance on global motion tasks in migraine groups compared with control groups. Success on global motion tasks relies on local motion detection, integration of motion signals and discrimination between noise and signal (Dakin et al., 2005, *Vision Research*, *45*, 3027–3049). Previous research has shown no difference in local motion detection (Shepherd et al., 2012, *Cephalalgia*, *32*(7), 554–570), no difference in baseline internal noise levels in migraine (Tibber et al., 2014, *Investigative Ophthalmology & Visual Science*, *55*(4), 2539–2546), but a difference in multiplicative noise levels (Wagner et al., 2010). This increased noise could be the result of differences in temporal integration processes in migraine compared with controls. The motion streak effect depends on the ability to integrate a moving point stimulus over time, enabling orientation detection mechanisms to be used to determine direction of motion more accurately than would otherwise be possible (Geissler, 1999, *Nature*, *400*, 65–69). The current data show that thresholds for the motion streak are higher in migraine compared with control groups, both for vertical and horizontal motion, indicating possible differences in temporal integration processes. This has implications for other visual task performance in migraine groups, as well as increased susceptibility to discomfort from flickering stimuli for these individuals.

## How Images Draw the Eye: An Eye-Tracking Study of Composition

**Clare Kirtley**

University of Aberdeen, Aberdeen, UK

### 

In his instructional art book, the artist Andrew Loomis provided images and corresponding diagrams that indicated how the composition of the image should guide the viewer’s eye. Using these images, we examined whether participants would follow the suggested cues shown in the diagrams. Participants’ eyes were tracked as they viewed the images, allowing us to take measures of where they entered and exited the image, whether they attended to the focal part of the image, and what path they followed between these components. These measures could then be compared with Loomis’ suggestions to determine if the elements did indeed have the proposed influence. While viewers were attracted to the focal points and spent the most time examining these, they did not use the entry and exit points marked by Loomis, and the suggested viewing paths did not resemble those proposed by the artist. It appears that Loomis’ suggested elements of composition do not strongly influence viewers’ eye-movements, in agreement with Leder et al.’s (2004, *British Journal of Psychiatry*, *95*, 489–508) model of aesthetic experience, which proposes that the perceptual analysis of art is only a small and early part of the ongoing process when viewing drawings.

## Body Size and Childhood Memories of Size and Slope

**Helen E. Ross**

Department of Psychology, University of Stirling, Stirling, UK

### 

Anecdotal reports agree that when adults revisit childhood locations, buildings and spaces look smaller and slopes look flatter than their memories of the situation. The size effect can be explained if size is judged partly in relation to the viewer’s body size, which grows with age. The slope effect is more difficult to explain. Geographical slope is perceived through some combination of vestibular and postural information, viewing height, the angle of regard and the information within the visual image. The latter contains texture gradients, determined by the optical angle between the geographical slope and the line of regard. For a viewer of a given height, a steeper downhill slope gives a smaller optical angle and a steeper uphill slope gives a larger optical angle. With a greater viewing height, the optical angle increases for both downhill and uphill views; so a taller person should perceive downhill slopes as flatter than a smaller person, but uphill slopes as steeper. Similarly, distance foreshortening might make downhill slopes appear flatter to an adult than a child, but uphill slopes steeper. Geometrical models cannot explain the flattening of both downhill and uphill slopes with viewing height. It is likely that leg length is important, because it affects the difficulty of traversing the terrain. It is possible that perceived difficulty influences the perception of downhill slopes (Proffitt et al., 2008, *Perception*, *37*, 321–323), but there is little evidence regarding uphill slopes. Predictions from different theories will be discussed.

## Does ‘Illusory’ Motion Pop-Out?

**Ian Thornton**

Department of Cognitive Science, University of Malta, Malta

**Suncica Zdravkovic**

University of Novi Sad, Serbia

### 

Akiyoshi Kitaoka has produced a number of very compelling ‘illusory motion’ displays, many directly descended from the classic peripheral drift illusion (Fraser & Wilcox, 1979). Modelling and imaging studies have long implicated the role of early visual mechanisms in these effects. Here, we address this question using a standard visual search task. In Experiment 1, participants searched for an illusory motion target amongst a variable number of distractors (set sizes: 2, 3, 4) organised in a 2 × 2 grid. The illusion target was custom made, a cross between Kitaoka’s ‘Rollers’ and ‘Rotating Snakes’ illusions, and gave a compelling impression of rotation. The distractors used exactly the same elements, but configured to eliminate any impression of motion. Average target present search slopes were completely flat, suggesting automatic capture of attention or pop-out. In Experiment 2, we increased the set size (4, 5, 6) and used a circular search array to control for eccentricity effects, again finding flat average search functions. Finally, in Experiment 3, we used high-pass and low-pass versions of the same stimuli – manipulations that eliminated motion but controlled for configuration differences between targets and distractors – finding slow, serial search in both cases. In short, illusory motion does appear to pop-out, providing clear psychophysical support for the role of early visual processes.

## Falling Waters and Rising Rocks – The Pursuit of an Enigma

**Nicholas J. Wade**

University of Dundee, Dundee, UK

### 

Robert Addams described the waterfall illusion following observation of the Falls of Foyers in 1834. The first enigma is as follows: Why was it described so late in the history of vision? It could be that maintaining a steady eye position while observing a moving stimulus required experimental and analytical approaches that were introduced in the early 19th century. Why is it called the waterfall illusion when the motion is seen in the rocks? This is the enigma of distinguishing adaptation from test. For Addams, apparent motion of the rocks was a consequence of unconscious pursuit eye movements when viewing descending water. Eye movements are almost always initially invoked for interpreting novel visual motion phenomena. This introduced a third enigma: An aftereffect of eye movements cannot be restricted to an isolated part of the visual scene. In 1880, Silvanus Thompson presented evidence against eye movement interpretations by demonstrating counter-rotating motion aftereffects (MAEs) seen simultaneously. Furthermore, John Aitken in 1878 reported that no MAE followed motion of the whole visual field (by rotating a striped cylinder around the head) but apparent body rotation (vection) did. Subsequent interpretations of the MAE were based on physiological processes (like adapting motion detectors), but there remain problems with these. Relative visual motion is required in the adapting stimulus and the test must stimulate both adapted and unadapted retinal regions. Thus, the stationary rocks are as vital as the falling waters to produce the waterfall illusion.

## Divided Attention Effects Are Larger for Change Detection Than for Simple Detection

**James C. Moreland, John Palmer and Geoffrey M. Boynton**

Department of Psychology, University of Washington, Washington, USA

### 

Studies of divided visual attention have largely depended on detection and search tasks because they minimize the role of memory and decision. Recently, the change detection paradigm has been used to study divided attention and the results interpreted as due to perception. Instead, might the divided attention effects in change detection be ‘inflated’ by memory and decision? To consider this, we compare divided attention effects for change detection and simple detection. Subjects were presented two intervals of dynamic 1/f noise patches on the left and right side of fixation and were cued beforehand to attend to either one side (selective attention) or both sides (divided attention). For the change detection task, Gabor patches were embedded in the noise on both sides, in both intervals, with a 50% chance of a 90° change in orientation from the first interval to the second. The probability of change was independent across sides. For the simple detection task, Gabor patches appeared in each side with 50% probability and subjects were asked to detect the presence of the patch. For the change detection task, subjects performed worse in the divided attention condition compared with the selective attention condition. However, for the simple detection task, there was almost no cost of divided attention compared with selective attention. In summary, for nearly identical stimuli, we show that the magnitude of divided attention effects depends strongly on the task. Ongoing studies examine whether these task effects depend only on perception or also on memory and decision.

## Double Cueing in the Posner Task

**Xuhong Liu**

School of Psychology, University of Glasgow, Glasgow, UK

**Gijsbert Stoet**

University Leeds-Beckett, Leeds, UK

**Martin Lages**

School of Psychology, University of Glasgow, Glasgow, UK

### 

In the original Posner paradigm (Posner & Cohen, 1984, *Attention and Performance X: Control of Language Processes*, *32*, 531–556), participants are asked to quickly respond to a visual target preceded by a cue at the same or a different location. If the stimulus onset asynchrony (SOA) between cue and target is short. participants typically respond quicker to a target cued by a stimulus at the same location than a different location (facilitation). For longer SOAs, participants respond more slowly to a target cued at the same location compared with a different location (Inhibition of Return [IOR]). Many studies have investigated facilitation and IOR but the dependency between the opposing effects is not well understood and facilitation is not always observed (e.g. Colzato et al., 2012, *Neuropsych*, *50*(1), 98–103). Here, we employed two successive cues to test whether an additional cue with different SOAs would systematically modulate facilitation and IOR. We also included randomly intermixed trials with single-cueing as a control. Results of *N* = 20 participants (aged 20.8 ± 2.5 years) confirmed facilitation at SOA 100 ms and IOR at 800 ms for single cueing. For double cueing, we found robust IOR for cues at 100 + 800 ms but very little facilitation for 100+100 ms. Interestingly, if the two cues appeared at the same location at 800+100 ms, then IOR was eliminated. The results suggest that double cueing at the same location can abolish IOR regardless of the target location.

## Transient Temporal Properties of Mirror-Symmetry Perception in Human Vision

**Rebecca J. Sharman and Elena Gheorghiu**

Department of Psychology, University of Stirling, Stirling, UK

### 

Mirror symmetry is believed to be encoded by specialized visual mechanisms. Here, we investigate transient temporal aspects of mirror-symmetry perception by examining how symmetric pattern elements are combined over time. Stimuli consisted of two successively presented images. There were five stimulus conditions: (a) symmetric pattern followed by noise pattern (whole condition); (b) left and right halves of the symmetric and noise patterns presented with temporal delay (delayed halves condition); (c) each pattern containing 50% of the symmetric pairs; (d) the same as condition (c), but the left and right halves presented with temporal delay (delayed 50% of the symmetric-pairs condition); and (e) noise and symmetry presented together as one single static pattern (static condition). We varied the presentation duration between 23.5 ms and 294 ms and the presentation order of the images. We measured symmetry detection thresholds using a 1AFC procedure. Participants indicated whether the stimulus was symmetric or not. Analysis of the slopes of the psychometric functions for each condition revealed that (a) in the delayed conditions, symmetry could only be detected for durations shorter than 120 ms per image; (b) in the whole condition, backward masking occurred for durations of less than 50 ms, while with forward masking, symmetry detection improved with duration; and (c) duration had no effect in the 50% of the symmetric-pairs condition. Symmetry detection mechanisms can compute cross-correlation between pattern elements across the symmetry axis up to delays of 120 ms and duration has less effect when symmetry is present in both intervals (50% of the symmetric-pairs) not just one (whole).

This research was supported by a Wellcome Trust Investigator grant (WT106969/Z/15/Z) given to EG.

## Zoomlens, Splitting, Doughnut: Exploring the Flexibility of Spatial Selective Attention in Vision

**Søren K. Andersen**

University of Aberdeen, Aberdeen, UK

### 

Much research on covert spatial attention has focussed on situations in which attention is directed to stimuli in the left or right visual fields. However, such situations may not be very representative of natural vision, as one would often directly fixate (overtly attend) such stimuli rather than attend them covertly. A different mode of covert spatial attention, which cannot easily be substituted by eye movements, is described by Eriksen’s Zoomlens model, which considers the trade-offs between spreading attention widely and focussing it narrowly. Although the Zoomlens model has been highly influential, investigations of its neural underpinnings are limited. We investigated attentional modulation of stimulus processing in early visual areas using recordings of steady-state visual evoked potentials (SSVEPs). Participants were presented with three concentric rings of randomly moving dots. They were cued to attend to different combinations of one, two or all three rings on different trials in order to detect brief coherent motion intervals in the cued rings. Consistent with the Zoomlens model, attentional modulation of SSVEP amplitudes was largest when attention could be focused narrowly on only one stimulus. SSVEP amplitudes also revealed that participants were able to divide attention or shape it like a doughnut (i.e. ignore stimuli in the centre). Surprisingly, the magnitude of attentional modulation was determined largely by the number of stimuli attended rather than their spatial layout. Thus, while supporting key ideas of the Zoomlens model, our data also suggested that attentional selection was more object-based than spatial.

## Two Types of Flanker Interference?

**Leili Soo, Ramakrishna Chakravarthi and Søren K. Andersen**

University of Aberdeen, Aberdeen, UK

### 

Nearby irrelevant stimuli interfere with the processing of relevant information. Such flanker interference has been studied both in the context of object recognition, where nearby flankers lead to crowding (Bouma, 1970, *Nature*, *226*, 177–178), and in attention research, where response incongruent flankers can lead to a drastic increase in reaction times (Eriksen & Eriksen, 1974, *Perception & Psychophysics, 16*(1), 143–149). The theories derived from these results differ vastly between the subfields: while crowding is considered a purely visual phenomenon, flanker congruency effects are interpreted in terms of high-level attentional selection. Are both phenomena really that separate or are they more closely related than the literature would suggest? To answer this question, we compared the magnitude of crowding and flanker congruency effects as a function of target-flanker distance. Crowding was assessed through the accuracy with which briefly presented targets in the visual periphery could be identified. Flanker congruency effects were assessed as the reaction time difference between congruent and incongruent flankers. Both crowding and flanker congruency effects increased with decreasing flanker distance and both exhibited a similar dependency on flanker distance. Thus, both types of flanker interference can be observed under the same circumstances and might thus be more closely related than suggested by the previous literature.

## Crowding Does Not Explain Numerosity Underestimation

**Ramakrishna Chakravarthi**

School of Psychology, University of Aberdeen, Aberdeen, UK

**Marco Bertamini**

Institute of Psychology, Health and Society, University of Liverpool, Liverpool, UK

### 

Humans have the remarkable ability to estimate the number of objects in a visual scene without counting. Further, it has been well documented that when elements cluster together, they are underestimated. The crowding hypothesis suggests that objects that are close together crowd each other, that is, render their identities unreportable. This crowding process is said to ‘texturise’ the objects, which explains the underestimation. Nevertheless, evidence for this hypothesis is circumstantial. Here, we directly tested the crowding hypothesis by assessing both estimation and crowding on the same configurations of oriented rectangles (varied between 15 and 33). We manipulated the spacing and similarity (contrast polarity) between the objects. Spacing dramatically modulated numerosity reports, but similarity did not. In contrast, surprisingly, neither spacing nor similarity modulated orientation discrimination. To determine if the failure to observe such modulations in crowding was a result of long-range grouping between objects, we included a standard crowding condition, where a target was surrounded by just four flankers. We replicated the lack of effects when a large number of objects were present but observed the usual effects of spacing and similarity in the standard crowding setup. Interestingly, in the latter, similarity had a stronger effect on identification than spacing. Taken together, the effects of spacing and similarity in the estimation task were markedly different from the effects in the crowding task. These results are incompatible with a crowding account of numerosity underestimation.

## Theta Oscillations Are Associated to Time Representations in Visual Memory

**Oana Alina Iosif, Fren Smulders, Peter de Weerd, Vincent van de Ven**

Department of Cognitive Neuroscience, Maastricht University, Netherlands

### 

Previous studies have proposed different mechanisms for how time is represented in memory. The involvement of different brain areas in memory of time has also been investigated, with the hippocampus, motor cortex and even visual areas proposed to play a role. Distributed low-frequency oscillations, in particular, in the theta range have also been suggested to be involved in memory formation and time perception. We investigated how time is represented in visual memory. Using noninvasive electroencephalography in human participants, we measured the neural activity evoked by visual stimuli in a paired-associate learning task. During learning, each pair, consisting of two abstract images, was learned with one of two possible implicit waiting times between stimuli. During testing, the pairs were presented with either the learned or the alternative waiting time. For pairs tested with the longer waiting time, we found a significant difference in the power of the theta frequency range, depending on the implicitly learned waiting time. In case learn and test waiting times did not match, there was a marked drop in power following the learned time point for the appearance of the visual stimulus. The difference between conditions was significant at parietal and occipital sites. This evidence shows that implicit time information can be encoded in associate memory as differences in oscillatory activity in the theta frequency range.

## Factors Influencing Serial Dependencies in Visual Perception

**Fraser Aitken and Justin Ales**

School of Psychology and Neuroscience, University of St. Andrews, St. Andrews, UK

### 

Serial dependence is a phenomenon in which people’s perceptions of current stimuli values depend on previously seen stimuli. However, it is still poorly understood which aspects of a stimuli affect the amount of serial dependence observed. One theory is that the visual system makes an estimate of what is in the environment based on fixed weighting of past and current stimuli values. Another explanation is that the visual system weights current and past stimuli values adaptively. When stimuli are clear, more weight is attached to new values. In contrast, in unclear conditions, more weight is attached to past values. Here, we test both models by using a paradigm that has previously shown serial dependency in the perception of orientation (Fischer & Whitney, 2011). In our experiment, we used a similar orientation judgment task in which participants viewed randomly oriented Gabors embedded in noise and then indicated the stimulus orientation by adjusting a line to match. To assess whether the amount of serial dependence depends on stimulus visibility, we manipulated the contrast of our stimuli (5%, 10% and 20%). We found that increasing the visibility increased participants’ accuracy in judging orientation. However, in contrast to Fischer and Whitney (2011), participants failed to show evidence of serial dependency for any contrast level. It is unclear why the results were discrepant. It is possibly due to the lower contrasts we used or larger range of orientations we tested. Therefore, the question as to what determines serial dependency is still open.

## Short-Term Visual Memories in Healthy Ageing

**Karin S. Pilz and Ian Hawes**

School of Psychology, University of Aberdeen, Aberdeen, UK

### 

The perception of motion and visual change relies on the integration or fusion of features in the visual world. Short-term visual memory is critical to this process to temporarily store the visual information prior to integration. Verniers, a pair of vertical bars that are spatially offset in the horizontal direction, are commonly used to assess feature fusion. When two verniers of opposing offsets are presented in rapid succession at the same location, they fuse and are perceived as one vernier with an almost aligned vernier offset. Observers have no conscious access to the individual stimuli. Light masks can be used to study the temporal dynamics of feature fusion, and it has been shown that unconscious feature fusion can last up to five times longer than the physical stimulus duration. Here, we studied the effects of healthy ageing on visual short-term memories. In a first step, we successively presented two verniers that were individually adjusted so that participants were unable to determine the offset direction of the perceived vernier. In a second step, we then presented light masks from −100 to 200 ms around stimulus onset to assess the duration of feature fusion. Confirming previous studies, the mask affected fusion from −100 to 120 ms around stimulus onset for younger adults. For older adults, however, fusion was only affected when the light mask was presented before but not when it was presented after stimulus onset. These results indicate that short-term visual memories are altered in healthy ageing.

## Does Self-Prioritisation Affect Perceptual Processes?

**Josephine Reuther and Ramakrishna Chakravarthi**

University of Aberdeen, Aberdeen, UK

### 

Recent studies have employed newly formed associations between identities (e.g. you, friend and stranger) and geometric shapes (e.g. circle, triangle and square) to study effects of self-prioritisation in the perceptual domain. Such studies have argued that objects that are associated with the self are perceptually enhanced. However, an alternative account is that the formation of such associations could itself be influenced by self-prioritisation, namely the self-reference effect in memory. That is, the effects that have been attributed to *perceptual* changes could instead be due to differences in *memory* introduced during the formation of shape-identity associations. This hypothesis was tested in two experiments. We found that even extended practice of shape-label associations, aimed at reducing memory differences among the shape-identity associations, failed to counteract the self-prioritisation effects. This might suggest that memory differences do not drive these effects. On the other hand, it could also suggest that a single training session is not sufficient to equate for differences in association strength between different shape-identity pairs. In a second experiment, we artificially introduced memory differences for associations between shapes and novel nonwords and found that behavioural response patterns resembled those observed in self-prioritisation studies. Taken together, these findings cannot exclude the possibility that the reported perceptual effects are influenced by memory differences in association formation.
Note: These abstracts have not been edited and are printed as submitted.

